# Comparison of ocular surface assessment outcomes between healthy controls and patients with obstructive sleep apnea–hypopnea syndrome: a meta-analysis of the literature

**DOI:** 10.3389/fphys.2023.1163947

**Published:** 2023-05-05

**Authors:** Jian Sun, Jie He, Zongan Liang

**Affiliations:** ^1^ Department of Respiratory and Critical Care Medicine, West China Hospital, Sichuan University, Chengdu, Sichuan, China; ^2^ Department of Pulmonary and Critical Care Medicine, The First Affiliated Hospital of Chengdu Medical College, Chengdu, Sichuan, China

**Keywords:** meta-analysis, ocular surface, obstructive sleep apnea–hypopnea syndrome, floppy eyelid syndrome, apnea–hypopnea index

## Abstract

**Objective:** This meta-analysis aims to determine whether ocular surface alterations are associated with disease severity in patients with obstructive sleep apnea–hypopnea syndrome (OSAHS).

**Methods:** The protocol for this systematic review and meta-analysis was registered in PROSPERO. We conducted the search in six electronic databases (China National Knowledge Infrastructure, EMBASE, Cochrane Library, Web of Science, Wanfang, and PubMed) from since the construction of the databases to 30 December 2022. The standard mean difference (SMD) and correlation coefficients are reported as measures of the effect size in the presence of retrieved data. In addition, the random effects model or fixed effects model was used in a combined analysis. Stata 11.0 and R 3.6.1 were used for statistical analyses of the data.

**Results:** A total of 15 studies satisfied the inclusion criteria for this study. The prevalence of floppy eyelid syndrome (FES) and dry eye syndrome in patients with obstructive sleep apnea–hypopnea syndrome was 40 and 48%, respectively. In addition, the Schirmer 1 value and tear break-up time (TBUT) were remarkably reduced in patients with OSAHS when compared to that of the controls. The ocular surface disease index (OSDI) scores, Oxford corneal staining scores, and the rates of loss in the meibomian glands were elevated in patients with obstructive sleep apnea–hypopnea syndrome when compared to that of the controls, especially those with severe disease. Moreover, the Schirmer 1 value and tear break-up time exhibited a negative correlation with the apnea–hypopnea index (AHI), and the OSDI showed a positive association with the apnea–hypopnea index.

**Conclusion:** Patients with OSAHS had a greater prevalence of FES than the healthy controls. They also showed lower Schirmer 1 value and tear break-up time but had a higher OSDI, Oxford corneal staining scores, and rates of loss in the meibomian glands than the healthy controls.

**Clinical Trial Registration:** (https://www.crd.york.ac.uk/prospero/display_record.php?RecordID=392527).

## 1 Introduction

Obstructive sleep apnea–hypopnea syndrome (OSAHS) is a severe and potentially fatal sleep disease characterized by recurring apneic events and awakenings through all stages of sleep, leading to enhanced oxidative stress, sympathetic activity, and inflammatory response (Wang et al., 2020; Fiedorczuk et al., 2023). The most prominent feature of this condition is chronic intermittent hypoxia, which stimulates chronic inflammatory processes, attenuates antioxidant mechanisms, and increases the production of reactive oxygen products during the reoxygenation phase (Vaccaro et al., 1992; Vakil et al., 2018; Ma et al., 2019). Patients with OSAHS have an increased risk of cardiovascular diseases (CVDs), like hypertension, heart failure, and coronary heart disease (Kohler, 2015; Stansbury and Strollo, 2015). Oxidative stress, sympathetic activity, and systemic inflammatory reactions are linked to chronic intermittent hypoxia that might impact the ocular vasculature (Kohler and Stradling, 2010). In addition, OSAHS is linked to multiple ocular surface diseases, such as floppy eyelid syndrome (FES), meibomian gland dysfunction, and dry eye (Lin et al., 2022; Liu et al., 2022; Mavigok et al., 2022), which are in turn associated with elevated blood carbon dioxide levels due to prolonged intermittent hypoxia. This results in alterations in the hemodynamics which includes high nighttime variations in blood pressure and dilated and enlarged cerebral blood vessels. These variables interfere with the natural hemodynamics of the eye and ultimately lead to the onset and progression of a range of ocular surface diseases (Dhillon et al., 2007; Grover, 2010).

Ocular surface diseases damage the ocular surface structures and functions, thus directly affecting the visual function in patients, which further deteriorates their mental health and quality of life (Tomic et al., 2013). The common ocular surface diseases are floppy eyelid syndrome (FES), dry eye, keratoconus, and meibomian gland dysfunction (Brautaset et al., 2015; Kim et al., 2018; Phillips et al., 2019). Schirmer 1, tear break-up time (TBUT), ocular surface disease index (OSDI), intraocular pressure, meibomian gland (MG) loss rates, and corneal fluorescein staining are important indices to evaluate ocular surface disease. The most common dry eye diagnostic tests are Schirmer 1, TBUT, and OSDI (Abusharha et al., 2022). Schirmer 1 is considered the gold standard method for measuring tear production (Veloso et al., 2020), while TBUT is the traditionally used method to measure and assess tear-film stability in the clinic (Hwang et al., 2020). The OSDI is used to assess the symptoms associated with dry eyes (Alanazi et al., 2022), and it has been extensively used as the method of evaluating corneal injuries, particularly injuries to the epithelium, by staining injured corneal sites with fluorescein (Fukuda and Sasaki, 2012). Accurate measurements of the intraocular pressure (IOP) are the key to diagnosing and monitoring glaucoma (Lee et al., 2021). The quantification of the area of MG loss is of importance when assessing meibomian gland dysfunction (MGD) (Deng et al., 2022). MGD may contribute to evaporative dry eye and aqueous-deficient dry eye, according to the International Workshop on Meibomian Gland Dysfunction (Lin et al., 2017). Previous studies have reported that patients with OSAHS have higher OSDI scores and corneal fluorescein staining scores but lower TBUT and Schirmer value than do healthy controls. These associations suggest the compromised ocular surface characteristics in patients with OSAHS, which might increase the risk of developing dry eye syndrome (Acar et al., 2013; Karaca et al., 2016). However, the study by Gunes et al. (2023) reports that the AHI seems to be insignificantly related to Schirmer values and OSDI scores. The relationship between the AHI and ocular surface parameters have to be further investigated. The dysfunction of the lid glands is another significant factor contributing to the development of dry eye syndrome (Baudouin et al., 2016). Two previous studies have examined the possible involvement of the MG in OSAHS, demonstrating that patients with OSAHS, particularly those with a severe condition, often have MG atrophy (Karaca et al., 2019; Muhafiz et al., 2020). Recently, the assessment of ocular surface disorders using non-invasive screening has become more common. Therefore, ocular surface evaluation in patients with OSAHS should not be neglected. A previous meta-analysis conducted by Garcia-Sanchez et al. (2022) examined the association between ocular diseases and OSAHS. They reported that OSAHS increased the risk of glaucoma and diabetic retinopathy. Cheong et al. (2023) also revealed in a meta-analysis that patients with OSAHS have a considerably elevated risk of FES. Nevertheless, the aforementioned studies did not analyze the ocular surface characteristics in patients with OSAHS. In addition, no previous meta-analysis has explored dry eye prevalence in OSAHS. Dry eye, being one of the most prevalent ocular surface disorders, should be considered by clinicians when treating patients with OSAHS. In addition, the relationship between the relevant dry eye screening indicators and AHI requires further in-depth studying.

Consequently, this study aims at providing a more comprehensive meta-analysis of the existing data to compare the ocular surface assessment outcomes between healthy controls and patients with OSAHS. Furthermore, we analyze the relationship between ocular surface changes and severity of the condition in patients with OSAHS.

## 2 Materials and methods

This review conforms with the guidelines of the Preferred Reporting Items for Systematic Reviews and Meta-Analyses (PRISMA). The protocol of the review is also registered in PROSPERO (CRD 42023392527).

### 2.1 Search strategy

Six databases (China National Knowledge Infrastructure, Wanfang, Web of Science, Cochrane Library, EMBASE, and PubMed) were searched for all the relevant published literature from since the construction of the databases till 30 December 2022. We used the following strategy for searching free text: (“sleep apnea” OR “nocturnal hypoxia” OR “nocturnal hypoxemia” OR “OSA” OR “obstructive sleep apnea” OR “obstructive sleep apnea syndrome” OR “syndrome, obstructive sleep apnea” OR “obstructive sleep apnea” OR “sleep apnea, obstructive”) AND (“ocular surface”) AND (“trial” OR “cohort” OR “case-control” OR “observational” OR “longitudinal” OR “study” OR “cross-sectional”). We further conducted manual searches of the bibliographies of reviews and included studies but obtained no additional relevant records.

### 2.2 Study selection

Two authors (JS and JH) used the citation management system EndNote 20 to independently identify publications that met the criteria. After screening the potential articles using titles and abstracts, complete texts were screened for final decisions regarding inclusion and exclusion of articles. The research subjects of the literature were adults (age ≥18 years). All adult randomized controlled trials (RCTs) and observational studies reporting any connection between OSAHS and ocular surface alterations in patients compared to healthy controls were included. The respiratory disturbance index, apnea–hypopnea index (AHI), and clinical diagnoses of OSAHS were used to quantify the incidence and severity of the disease [e.g., International Classification of Diseases (ICD) diagnostic codes] (Bindi et al., 2022). The degree of severity of OSAHS was measured based on conventional standards. The following OSAHS severity criteria based on the AHI apply to adults: normal, AHI <5; mild, AHI 5–14; moderate, AHI 15–29; and severe, AHI ≥30 (McCann et al., 2009; Bitners and Arens, 2020). We also included academic dissertations, conference abstracts, and other forms of gray literature that satisfied the aforementioned requirements. However, experiments conducted using animal, reviews, case reports, and letters were not considered.

### 2.3 Data extraction

The data from each publication were extracted by three researchers (SJ, LZ, and HJ) and standardized into a common spreadsheet format that included all the relevant details, such as name of the first author, publication year, research design, setting, sample size, participant’s demographics, percentage of males, applicable exposures and interventions, outcomes, control variables, statistical methods, and ocular surface examination items.

### 2.4 Literature quality evaluation

Since the studies that satisfied the inclusion criteria were all observational, we assessed the potential for bias using the Newcastle–Ottawa Scale (Stang, 2010). Studies were evaluated based on their potential for bias and given a risk of bias rating of either high (<5 stars), moderate (5–7 stars), or low (≥8 stars).

### 2.5 Statistical analysis

The retrieved data were summarized and analyzed with the aid of the R (v. 3.6.1) and Stata (v. 11.0) statistical software programs. The standard mean difference (SMD) was used with a 95% confidence interval (CI) to describe the continuous variables after normalization. A meta-analysis was conducted using Spearman’s correlation coefficients (CORs) to probe the links between Schirmer 1, TBUT, OSDI, and AHI scores in patients with OSAHS. Spearman’s product-moment COR was not likely to be dependent on the sample distribution based on the standard error, which is often reliant on the significance of the rank COR. Fisher transformation was employed to make direct comparisons across all CORs. Afterward, the analysis was completed using the transformed values as input values before reverting them to CORs (Chen et al., 2013). The calculated effect size (small, ≤0.3; moderate, 0.3–0.5; and large, >0.5) was analyzed using Cohen’s criterion. Moreover, Spearman’s COR was employed to study the correlation between Schirmer 1, TBUT, OSDI, and AHI scores. In line with this explanation, the following formula has been cited by multiple research reports as a means of transforming Pearson’s COR to Spearman’s COR:*r* = 2 sin (*r*_s_ $\left.\frac{\pi }{6}\right)$ where *r* and *r*_*s*_ denote the CORs calculated using Pearson’s and Spearman’s methods, respectively (Wang et al., 2019). The heterogeneity of the data was analyzed by chi-square and Cochran’s Q tests. The degree of heterogeneity was evaluated with the I^2^ statistic (low heterogeneity was indicated by 25%, moderate by 50%, and high by 75%). The study heterogeneity was considered low when the I^2^ value was <50% and high when it was ≥50%. We used both fixed and random effects models to account for the possibility of perfect (zero) homogeneity across the studies.

We evaluated the possibility of publication bias and sensitivity analysis if more than 10 studies were included. As a part of the sensitivity analysis, individual studies were each removed to see how their results affected the overall effect size. To assess the existence of publication bias, we used Egger’s tests and linear regression.

## 3 Results

### 3.1 Retrieved and included articles in review

**FIGURE 1:**
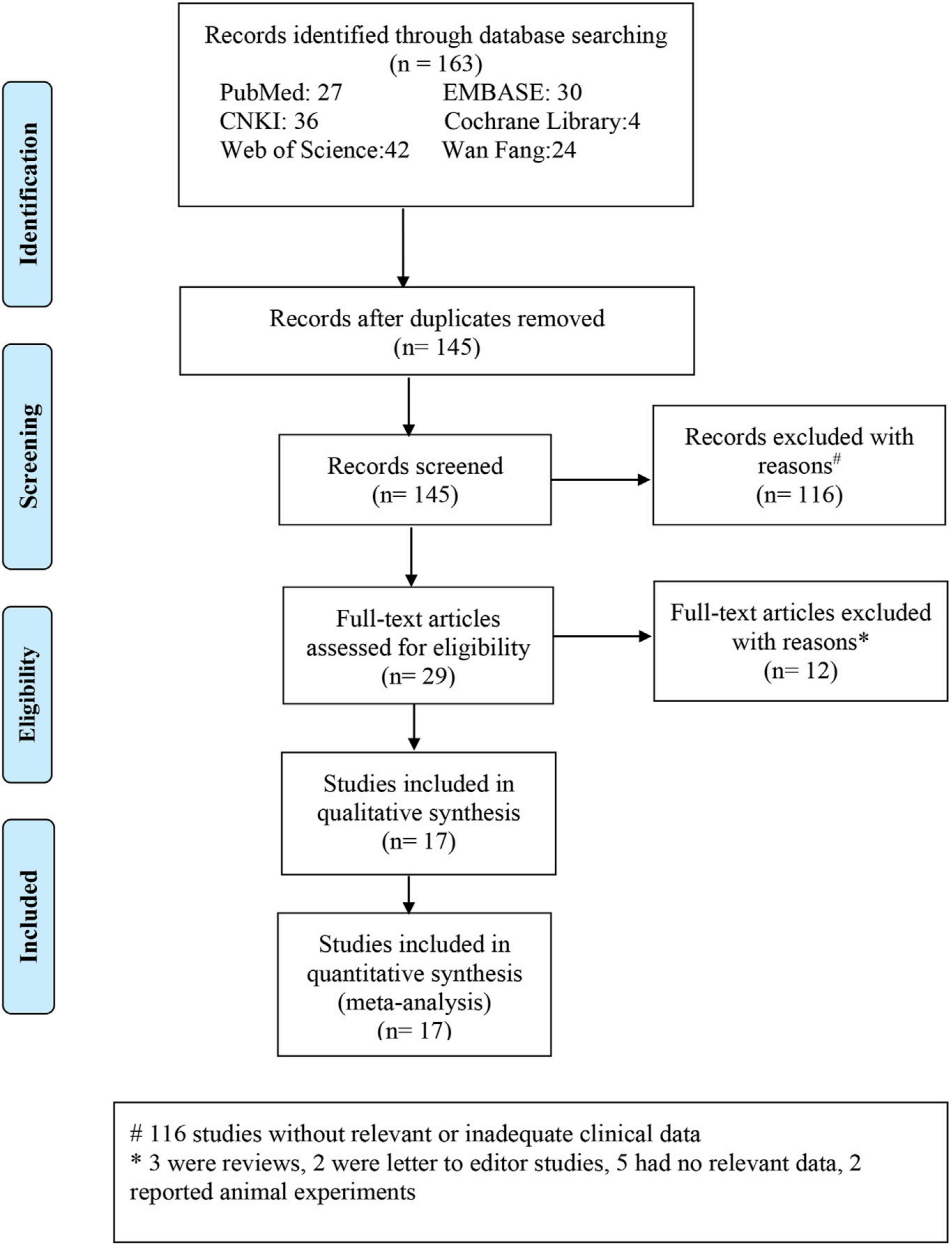
Process flowchart depicting the stages of selecting relevant literature and outcomes depending on the reporting items of the selected meta-analysis.

Overall, 163 research studies that were relevant to the topic were compiled from the databases. After the removal of duplicates, 145 articles were screened. After excluding 116 obviously irrelevant references while screening the abstracts and titles, the total number of studies was 29. After downloading the 29 publications, we examined the complete texts in detail. As per the inclusion and exclusion criteria, 12 publications were excluded. The following parameters were used to exclude these publications: three reviews, two letters to the editor, five lacked applicable data, and two were experiments on animals. Finally, 17 publications (Kadyan et al., 2010; Acar et al., 2013; Karaca et al., 2016; Fox et al., 2017; Liu and Gao, 2017; Acar et al., 2018; Karaca et al., 2019; Cristescu and Mihaltan, 2020; Muhafiz et al., 2020; Bi et al., 2022; Bonacci et al., 2022; Lin et al., 2022; Liu et al., 2022; Mavigok et al., 2022; Pu et al., 2022; Ulutas et al., 2022; Gunes et al., 2023) were included in the meta-analysis (Figure 1). For the patients with OSAHS, six studies (Acar et al., 2013; Karaca et al., 2019; Muhafiz et al., 2020; Lin et al., 2022; Liu et al., 2022; Ulutas et al., 2022) examined the incidence of FES and four studies examined the incidence of dry eye (Acar et al., 2018; Liu et al., 2022; Pu et al., 2022; Gunes et al., 2023) (Table 1). Sixteen articles (Kadyan et al., 2010; Acar et al., 2013; Karaca et al., 2016; Fox et al., 2017; Liu and Gao, 2017; Acar et al., 2018; Karaca et al., 2019; Cristescu and Mihaltan, 2020; Muhafiz et al., 2020; Bi et al., 2022; Bonacci et al., 2022; Lin et al., 2022; Mavigok et al., 2022; Pu et al., 2022; Ulutas et al., 2022; Gunes et al., 2023) compared the ocular surface assessment outcomes of the patients with OSAHS and healthy controls. Seven publications (Karaca et al., 2016; Cristescu and Mihaltan, 2020; Muhafiz et al., 2020; Lin et al., 2022; Liu et al., 2022; Pu et al., 2022; Gunes et al., 2023) reported a COR between Schirmer 1 and AHI scores (either Spearman’s or Pearson’s). Pearson’s or Spearman’s COR between TBUT and AHI scores was reported in six research studies (Karaca et al., 2016; Muhafiz et al., 2020; Lin et al., 2022; Liu et al., 2022; Pu et al., 2022; Gunes et al., 2023). Pearson’s or Spearman’s CORs were presented between OSDI and AHI scores in four publications (Karaca et al., 2016; Liu et al., 2022; Pu et al., 2022; Gunes et al., 2023). All the included studies examined adult participants only. The PRISM process flow for choosing and vetting publications from the literature is depicted in Figure 1. As shown in Table 1, the included studies provide basic information about their results.

**TABLE 1:** Characteristics of included studies.

First author	Year	Study design	Sample size		OSAHS diagnosis	Country	Age		Gender (male/female)	NOS	Ocular surface assessment method
			Case	Control			Case	Control			
Acar Ma	2013	CCS	60	26	PSG	Turkey	43.9 ± 11.5	46.7 ± 9	53/33	7	Schirmer, TBUT, OSDI, and corneal staining
Acar Mb	2013	CCS	72	26	PSG	Turkey	49.9 ± 9.3	46.7 ± 9	54/44	7	Schirmer, TBUT, OSDI, and corneal staining
Acar Mc	2013	CCS	122	26	PSG	Turkey	48.1 ± 10.5	46.7 ± 9	110/38	7	Schirmer, TBUT, OSDI, and corneal staining
Acar Ma	2018	CCS	12	14	PSG	Turkey	48.8 ± 4.8	46.8 ± 4.4	21/5	7	Schirmer, TBUT, and OSDI
Acar Mb	2018	CCS	16	14	PSG	Turkey	47.3 ± 6.1	46.8 ± 4.4	NA	7	Schirmer, TBUT, and OSDI
Acar Mc	2018	CCS	13	14	PSG	Turkey	49 ± 4.2	46.8 ± 4.4	27/3	7	Schirmer, TBUT, and OSDI
Bi XDa	2022	CSS	17	26	PSG	China	38.41 ± 13.76	41.58 ± 15.17	22/85	6	Schirmer, TBUT, OSDI
Bi XDb	2022	CSS	19	26	PSG	China	43.16 ± 13.37	41.58 ± 15.17	NA	6	Schirmer, TBUT, and OSDI
Bi XDc	2022	CSS	23	26	PSG	China	41.1 ± 12.55	41.58 ± 15.17	NA	6	Schirmer, TBUT, and OSDI
Fox Tpa	2017	CCS	33	35	PSG	America	53.2 ± 15.4	48.5 ± 16.3	25/43	8	Corneal staining
Fox Tpb	2017	CCS	70	35	PSG	America	52.7 ± 11.3	48.5 ± 16.3	55/50	8	Corneal staining
Fox Tpc	2017	CCS	63	35	PSG	America	55.5 ± 12.3	48.5 ± 16.3	58/40	8	Corneal staining
Gunes Ia	2022	CSS	23	29	PSG	Turkey	47.57 ± 10.52	42.86 ± 12.54	33/28	7	Schirmer, TBUT, and OSDI
Gunes Ib	2022	CSS	27	29	PSG	Turkey	47.26 ± 11.51	42.86 ± 12.54	30/22	7	Schirmer, TBUT, and OSDI
Gunes Ic	2022	CSS	27	29	PSG	Turkey	51.26 ± 12.09	42.86 ± 12.54	38/18	7	Schirmer, TBUT, and OSDI
Karaca EEa	2016	CSS	15	50	PSG	Turkey	42.1 ± 10.8	46.9 ± 12.2	37/28	6	Schirmer, TBUT, OSDI, and IOP
Karaca EEb	2016	CSS	15	50	PSG	Turkey	52.6 ± 10.6	46.9 ± 12.2	41/24	6	Schirmer, TBUT, OSDI, and IOP
Karaca EEc	2016	CSS	20	50	PSG	Turkey	49.1 ± 9.2	46.9 ± 12.2	45/25	6	Schirmer, TBUT, OSDI, and IOP
Karaca I	2019	CSS	36	24	PSG	Turkey	50.8 ± 8.3	47.9 ± 10.5	22/5	7	Schirmer, TBUT, OSDI, and corneal staining
Lin PWa	2022	CSS	53	26	PSG	China	38 (32.8–44.5)	36.5 (31–49)	NA	8	Schirmer, TBUT, OSDI, and corneal staining
Lin PWb	2022	CSS	42	26	PSG	China	40.5 (32–50)	36.5 (31–49)	39/40	8	Schirmer, TBUT, OSDI, and corneal staining
Lin PWc	2022	CSS	60	26	PSG	China	43 (35–48.5)	36.5 (31–49)	36/32	8	Schirmer, TBUT, OSDI, and corneal staining
Liu M	2017	CCS	63	44	PSG	China	42.1 ± 11.1	43.8 ± 8.6	37/19	6	TBUT and OSDI
Liu SH	2022	CSS	103	NA	PSG	China	38.1 ± 7.5	NA	96/7	6	Schirmer, TBUT, and OSDI
Mavigok E	2022	CCS	31	30	PSG	Turkey	51.09 ± 10.89	47.13 ± 15.49	70/24	7	Schirmer, TBUT, OSDI, and IOP
Muhafiz E	2020	CCS	32	27	PSG	Turkey	45.06 ± 12.95	47.73 ± 7.39	41/19	6	Schirmer and TBUT
Pu Q	2022	CCS	125	125	PSG	China	54.12 ± 12.99	53.72 ± 13.03	65/21	7	Schirmer, TBUT, OSDI, and corneal staining
Bonacci E	2022	CCS	35	37	PSG	Italy	10.31 ± 3.6	11.1 ± 3.41	30/42	7	IOP
Kadyan	2010	CCS	89	26	PSG	England	55.75 ± 10.97	55.3 ± 10.7	92/23	8	IOP and TBUT
Ulutas HG	2022	CSS	47	47	PSG	Turkey	45.77 ± 9.6	44.26 ± 8.54	154/96	7	Schirmer, TBUT, OSDI, and IOP
Cristescu TR	2020	CS	65	39	PSG	Romania	34–98	36–94	NA	7	Schirmer and IOP
					STOP-BANG						

OSAHS, obstructive sleep apnea hypopnea syndrome; NOS, Newcastle–Ottawa Scale; a, mild; b, moderate; c, severe; NA, not available; OSDI, Ocular Surface Disease Index; TBUT, tear film break-up time; IOP, intraocular pressure; CCS, case–control study; CSS, cross-sectional study. PSG: polysomnography STOP-BANG: the snoring, tiredness, observed apnea, high blood Pressure (STOP)-body mass index, age, neck circumference, and gender (BANG) questionnaire.

### 3.2 Prevalence of FES in patients with OSAHS

**FIGURE 2:**
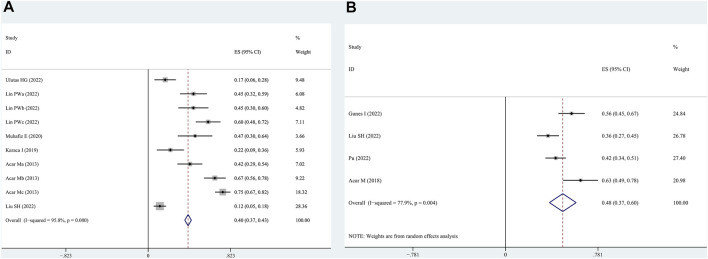
Prevalence of floppy eyelid syndrome (FES) and dry eye syndrome in patients with OSAHS as determined by the random effects model. **(A)**: FES; **(B)**: dry eye syndrome.

Six articles reported the number of patients with OSAHS having combined FES. Thus, these articles, involving 627 patients with OSAHS and 286 patients who were FES-positive, were subjected to a combined analysis. The meta-analysis showed that the total prevalence rate of FES in the patients with OSAHS was 40% (95% CI, 0.37–0.43; *p* < 0.001; Figure 2A).

### 3.3 Prevalence of dry eye in patients with OSAHS

Four articles reported patients with OSAHS having comorbid dry eye. These articles, involving 346 patients with OSAHS and 159 patients with dry eye, were subjected to a combined analysis. The meta-analysis results illustrate that the total prevalence of dry eye in the patients with OSAHS was 48% (95% CI, 0.37–0.60; *p* < 0.001; Figure 2B).

### 3.4 Differences in Schirmer 1 values between patients with OSAHS and healthy subjects

**FIGURE 3:**
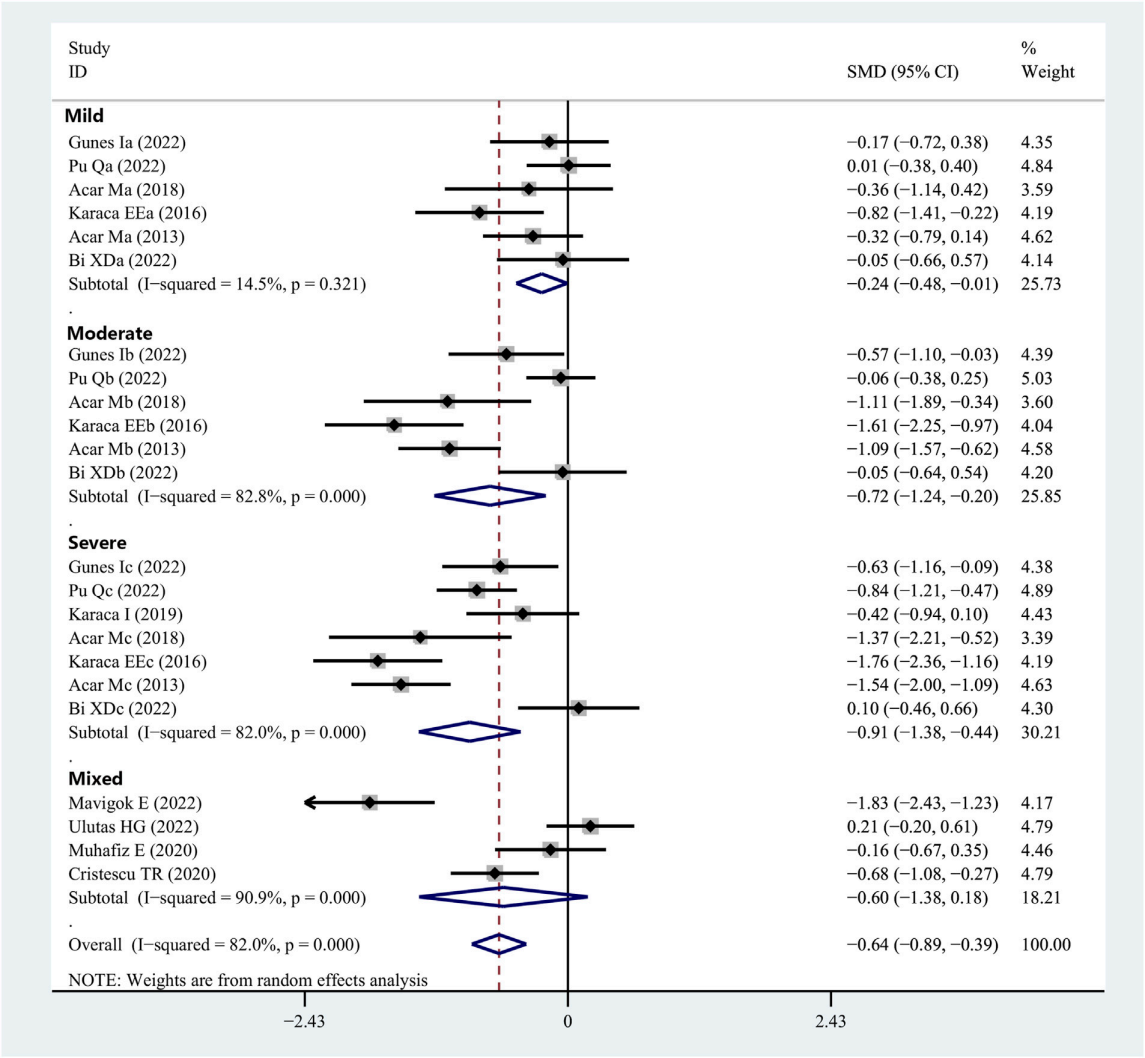
Forest plot depicting the SMD and its 95% CI for Schirmer 1 value in patients with integral OSAHS when compared with the healthy controls in the meta-analysis. SMD, standard mean difference; CI, confidence interval; and OSAHS, obstructive sleep apnea–hypopnea syndrome.

Eleven studies provided Schirmer 1 data for patients with OSAHS and normal controls. Patients with OSAHS had lower Schirmer 1 values than the normal controls (SMD, −0.64; 95% CI, −0.89 to −0.39; *p* < 0.001; I^2^, 82%). The influence of the severity of illness on the Schirmer 1 values of the patients was investigated using subgroup analysis, which was completed using the disease severity parameter. The subgroup analysis results indicated that the participants in the groups with mild (SMD, −0.24; 95% CI, −0.48 to −0.01; *p* = 0.044), moderate (SMD, −0.72, 95% CI, −1.24 to −0.20; *p* = 0.006), and severe (SMD, −0.91; 95% CI, −1.38 to −0.44; *p* < 0.001) OSAHS all exhibited lower Schirmer 1 values than the healthy participants. In addition, the Schirmer 1 values showed a more significant decrease with increasing disease severity (Figure 3; Table 2).

**TABLE 2:** Ocular surface parameter comparing between the control and case groups for OSAHS.

Ocular surface parameter (study = n)	SMD (95% CI)	*p*-value	I^2^(%)	Ph
Schirmer 1				
Mild (Kohler, 2015)	−0.24 (−0.48 to 0.01)	0.044	14.5	0.321
Moderate (Kohler, 2015)	−0.72 (−1.24 to 0.20)	0.006	82.8	<0.001
Severe (Stansbury and Strollo, 2015)	−0.91 (−1.38 to 0.44)	<0.001	82.0	<0.001
TBUT				
Mild (Stansbury and Strollo, 2015)	−0.24 (−0.48 to 0.01)	0.055	31.8	0.185
Moderate (Stansbury and Strollo, 2015)	−0.91 (−1.31 to 0.51)	<0.001	75.5	<0.001
Severe (Kohler and Stradling, 2010)	−1.07 (−1.37 to 0.76)	<0.001	62.3	0.010
OSDI scores				
Mild (Stansbury and Strollo, 2015)	0.62 (−0.02 to 1.25)	0.056	89.2	<0.001
Moderate (Stansbury and Strollo, 2015)	1.33 (0.46–2.19)	0.003	94.2	<0.001
Severe (Stansbury and Strollo, 2015)	1.75 (0.66–2.84)	0.002	96.1	<0.001
Oxford corneal staining scores				
Mild (Vaccaro et al., 1992)	−0.03 (−0.38 to 0.31)	0.850	57.9	0.068
Moderate (Vaccaro et al., 1992)	0.28 (−0.29 to 0.84)	0.343	86.6	<0.001
Severe (Vaccaro et al., 1992)	0.47 (0.07–0.86)	0.021	72.2	<0.001
Intraocular pressure (Vakil et al., 2018)	0.43 (−0.03 to 0.89)	0.64	80.7	<0.001
Rates of loss in the meibomian glands (Kohler and Stradling, 2010)	0.69 (0.25–1.13)	0.002	89.7	<0.001

Ph, P_heterogeneity_; OSDI, Ocular Surface Disease Index; TBUT, tear film breakup time.

### 3.5 Differences in TBUT between OSAHS patients and healthy subjects

**FIGURE 4:**
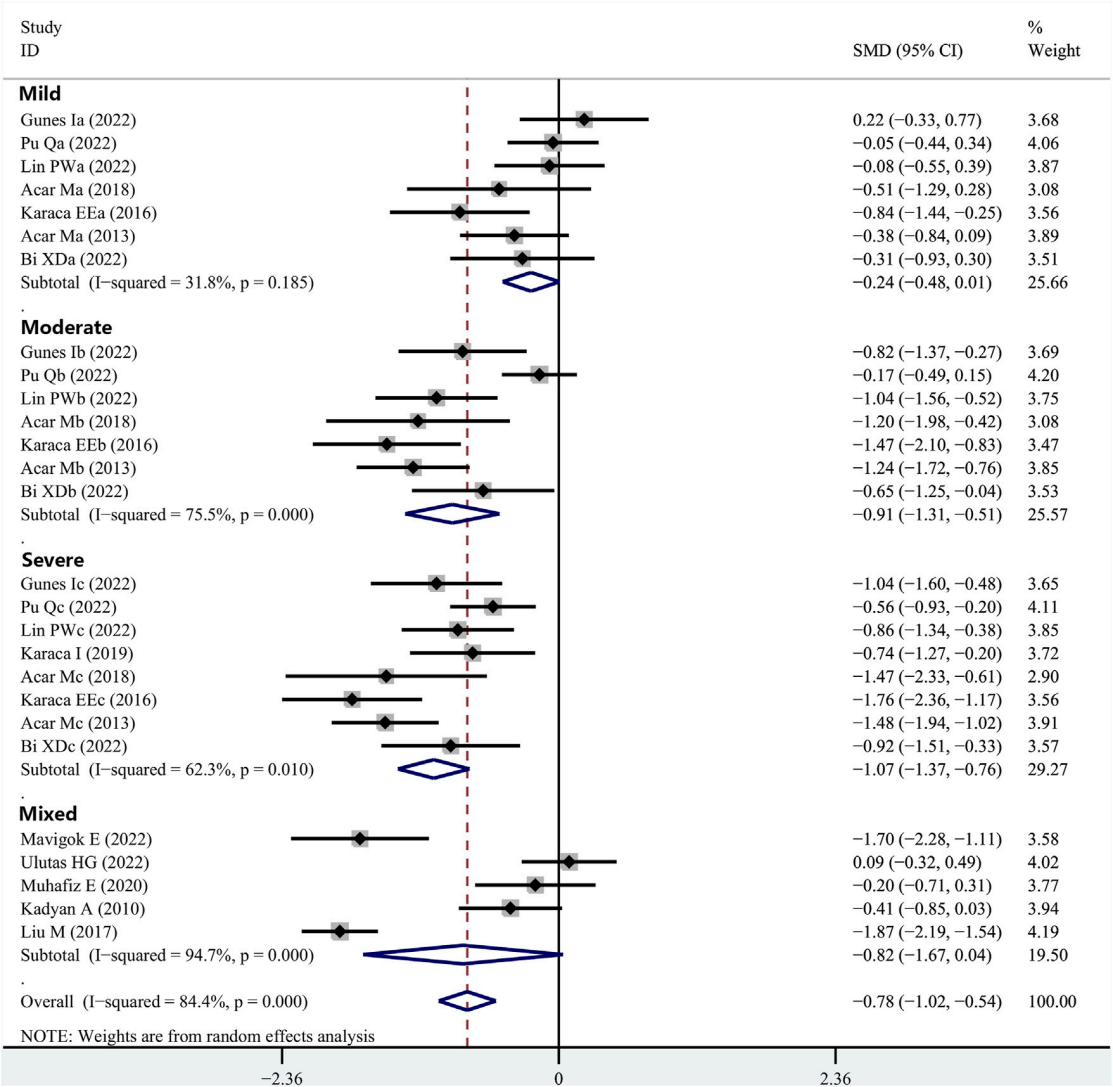
Comparison of the TBUT values between the patients with integral OSAHS and controls, shown as a forest plot of the SMD and its 95% CI. SMD, standard mean difference; CI, confidence interval; OSAHS, obstructive sleep apnea–hypopnea syndrome; TBUT, tear break-up time.

Fourteen articles reported data on TBUT outcomes in patients with OSAHS and normal controls. TBUT was shown to be substantially lower in the patients diagnosed with OSAHS than in the healthy participants (SMD, −0.78; 95% CI, −1.02 to −0.54; *p* < 0.001; I^2^, 84.4%). Subsequently, the disease severity was considered for the subgroup analysis. In the group of patients diagnosed with mild OSAHS, there was no significant difference in the TBUT values between the patients with OSAHS and healthy participants (SMD, −0.24; 95% CI, −0.48 to −0.01; *p* = 0.055). However, in both the moderate and severe OSAHS groups, the patients had lower TBUT than the healthy subjects (SMD, −0.91; 95% CI, −1.31 to −0.51; *p* < 0.001 and SMD, −1.07; 95% CI, −1.37 to −0.76; *p* < 0.001, respectively). In addition, the TBUT values showed a more significant decrease with increasing disease severity (Figure 4; Table 2).

### 3.6 Differences in OSDI scores between OSAHS patients and healthy subjects

**FIGURE 5:**
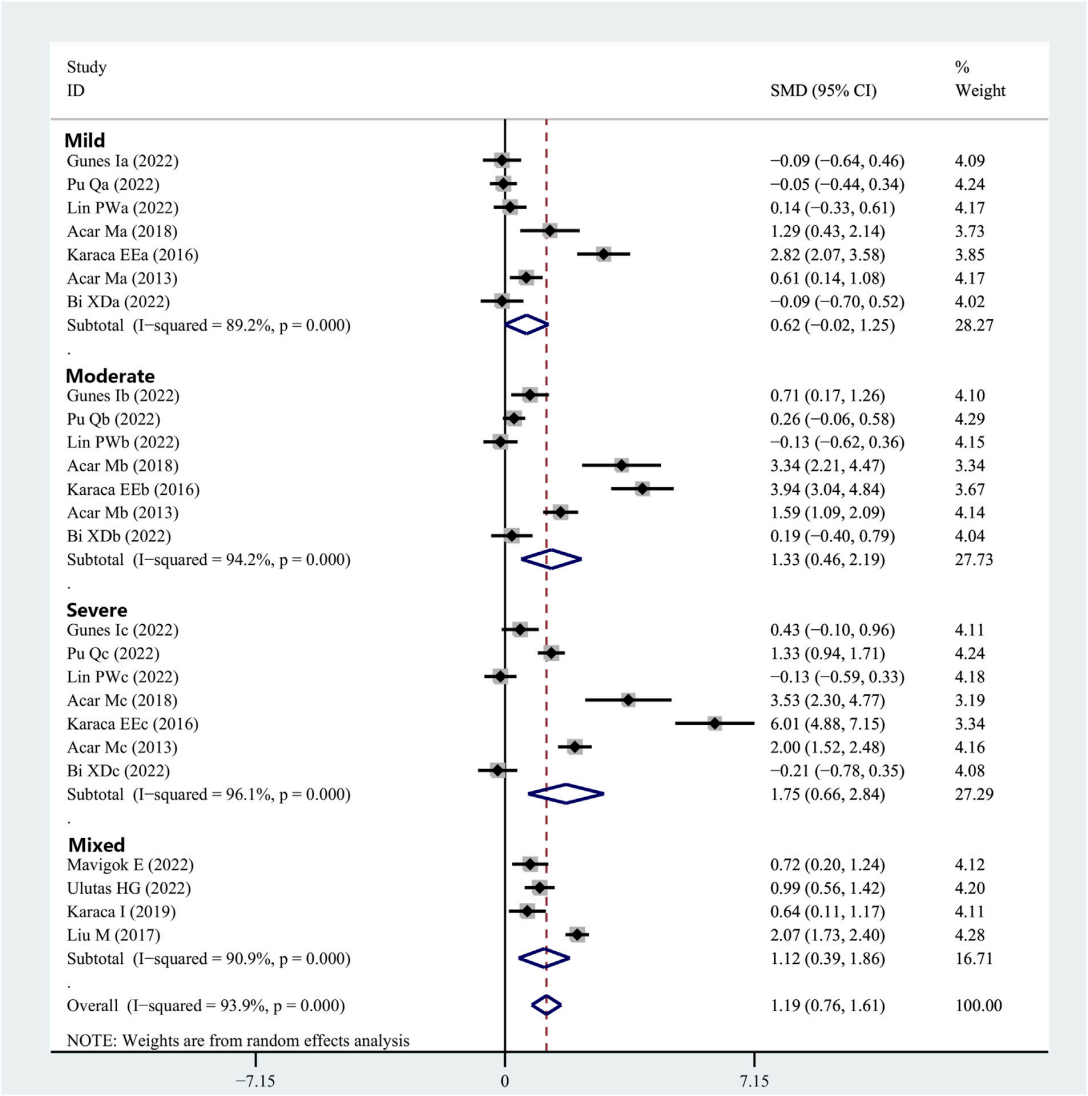
Meta-analysis of the SMD and 95% CI for the OSDI scores among the patients with integrated OSAHS and controls, shown as a forest plot. SMD, standard mean difference; CI, confidence interval; OSAHS, obstructive sleep apnea–hypopnea syndrome; OSDI, ocular surface disease index.

Eleven articles provided data on the OSDI scores in patients with OSAHS and healthy controls. The OSDI scores were substantially higher in the patients diagnosed with OSAHS than in the healthy controls (SMD, 1.19; 95% CI, 0.76–1.61; *p* < 0.001; I^2^, 93.9%). Subsequently, the severity of the illness was considered when performing the subgroup analysis. In the group of patients having mild OSAHS, there was no significant difference in terms of OSDI scores between the patients with OSAHS and healthy participants (SMD, 0.62; 95%, CI, −0.02 to 1.25; *p* = 0.056). However, in both the moderate and severe OSAHS groups, the patients had higher OSDI scores than the healthy subjects (SMD, 1.33; 95% CI, 0.46–2.19; *p* = 0.003 and SMD, 1.75; 95% CI, 0.66–2.84; *p* = 0.002, respectively). In addition, the OSDI scores showed a more significant increase with increasing disease severity (Figure 5; Table 2).

### 3.7 Differences in Oxford corneal staining scores between patients with OSAHS and healthy controls

Four articles provided data on the Oxford corneal staining scores for patients with OSAHS and healthy controls. The patients with OSAHS did not show a significant variation in their Oxford corneal staining scores when compared to the healthy controls (SMD, 0.31; 95% CI, −0.04 to 0.66; *p* = 0.081; I^2^, 83.8%). Subsequently, the severity of the illness was subjected to subgroup analysis. No significant difference was observed in the Oxford corneal staining scores between the patients with OSAHS and healthy controls in the groups with patients having mild and moderate OSAHS (SMD, −0.04; 95% CI, −0.52 to 0.43; *p* = 0.866 and SMD, 0.36; 95% CI, −0.46 to 1.18; *p* = 0.388, respectively). However, in the group with patients having severe OSAHS, the patients had higher Oxford corneal staining scores than did the healthy controls (SMD, 0.61; 95% CI, 0.21–1.01; *p* = 0.003; Table 2).

### 3.8 Differences in intraocular pressure between patients with OSAHS and healthy controls

Five studies provided data on the intraocular pressure in patients with OSAHS and healthy controls. There was no significant difference between the intraocular pressure in patients with OSAHS and the healthy controls (SMD, 0.43; 95% CI, −0.03 to 0.89; *p* = 0.064; I^2^, 80.7%; Table 2).

### 3.9 Differences in MG loss rates between patients with OSAHS and healthy controls

The comparison between the MG loss rates in patients with OSAHS and healthy controls was reported in three studies. The patients with OSAHS had a higher incidence of MG atrophy than the normal participants (SMD, 0.69; 95% CI, 0.25–1.13; *p* = 0.002; I^2^, 89.7%; Table 2).

### 3.10 Correlational meta-analysis of Schirmer 1, TBUT, OSDI, and AHI scores

**FIGURE 6:**
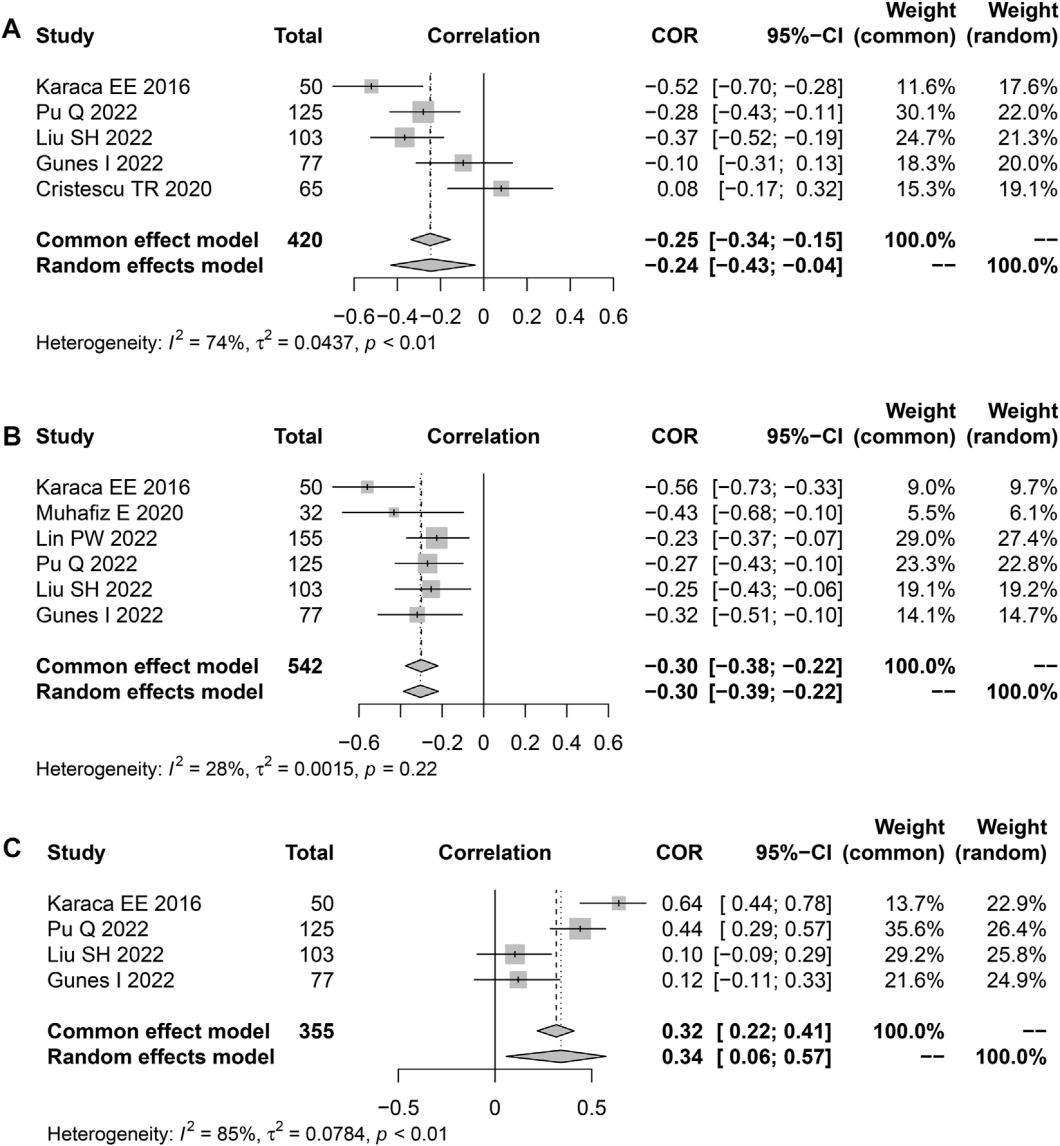
Funnel plot displaying the associations between the Schirmer 1, TBUT, OSDI scores, and AHI in terms of effect sizes. **(A)** Schirmer 1; **(B)** TBUT; and **(C)** OSDI scores. TBUT, tear break-up time; OSDI, ocular surface disease index.

Seven studies reported Spearman’s or Pearson’s CORs between Schirmer 1 and AHI scores (Table 3). The severity of OSAHS may be evaluated using the AHI score. The “meta” R soft package was used to conduct a meta-analysis on the findings of the Schirmer 1 and the AHI scores recorded from the patients with OSAHS. The results suggested an effect size of −0.24 (95% CI, −0.43 to 0.04; *p* = 0.019; I^2^, 74%) for the correlation between the Schirmer 1 values and AHI scores (Figure 6A). The correlation between TBUT and AHI scores was reported in six publications using either Spearman’s or Pearson’s CORs (Table 3). The results showed a correlation effect value of −0.30 between the TBUT and AHI scores (95% CI, −0.39 to −0.22; *p* < 0.001; I^2^, 28%; Figure 6B). The correlation between the OSDI and AHI scores was reported in four studies using either Spearman’s or Pearson’s COR (Table 3). The correlation effect value between the OSDI and AHI scores was 0.34 (95% CI, 0.06–0.57; *p* = 0.019; I^2^, 85%; Figure 6C).

**TABLE 3:** Correlation coefficients of included studies.

Author	Year	N	Cor. of Schirmer 1 *vs.* AHI	Cor. of TBUT *vs.* AHI	Cor. of OSDI scores *vs.* AHI
Karaca EE	2016	50	−0.56	−0.56	0.64
Muhafiz E	2020	32	−0.432	−0.432	
Lin PW	2022	155	−0.226	−0.226	
Pu Q	2022	125	−0.27	−0.27	0.44
Liu SH	2022	103	−0.253	−0.253	0.103
Gunes I	2022	77	−0.32	−0.32	0.12
Cristescu TR	2020	65	0.081		

### 3.11 Publication bias and sensitivity analysis

**FIGURE 7:**
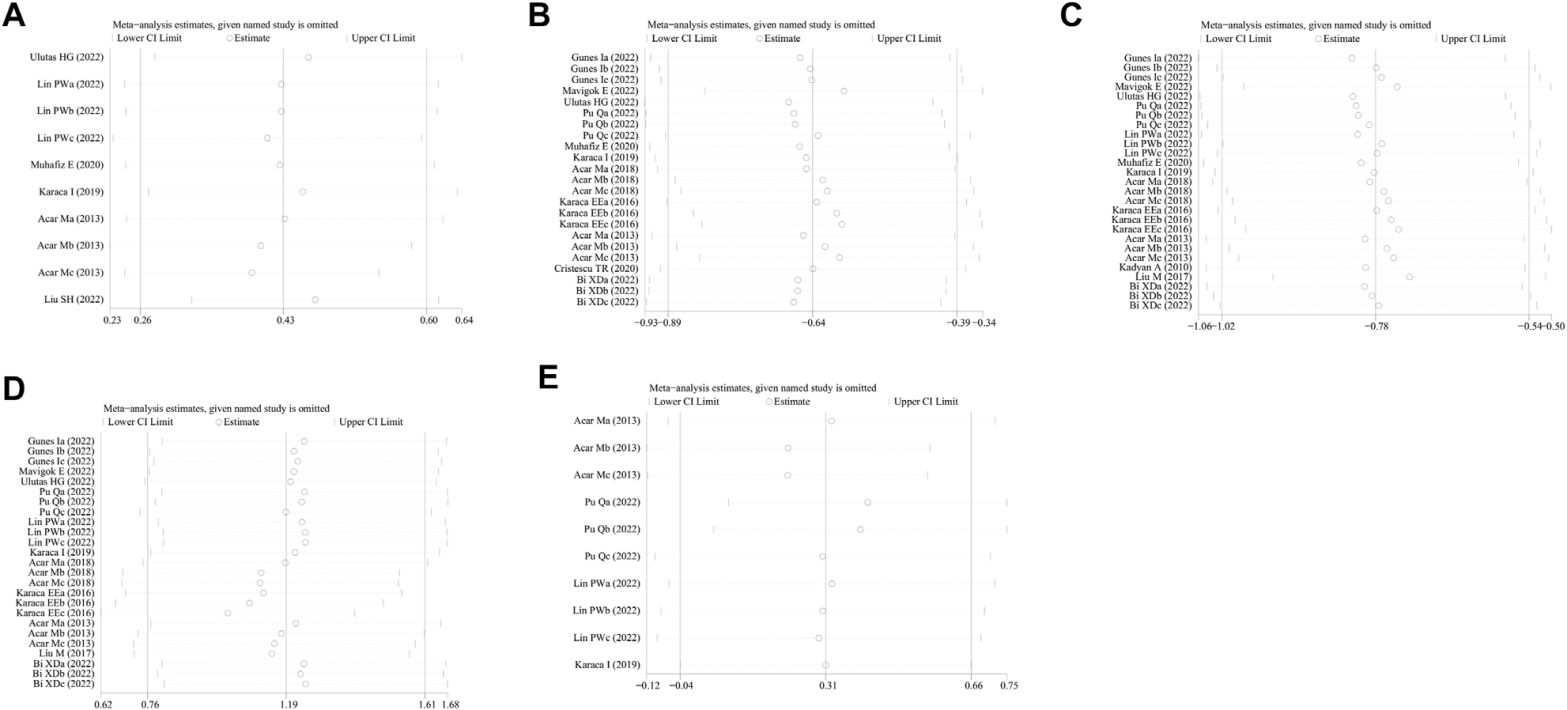
Sensitivity analysis of the influence of subsequent testing on the overall findings by systematically omitting specific studies. **(A)** Prevalence of FES; **(B)** Schirmer 1 value; **(C)** TBUT; **(D)** OSDI; and **(E)** Oxford corneal staining scores. FES, floppy eyelid syndrome; TBUT, tear break-up time; OSDI, ocular surface disease index.

**FIGURE 8:**
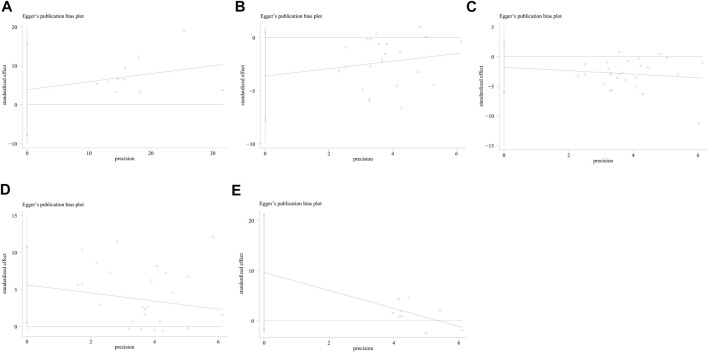
Publication bias of the included studies assessing the prevalence of laryngopharyngeal reflux in the patients with OSAHS was evaluated using funnel plots. **(A)** Prevalence of FES; **(B)** Schirmer 1 value; **(C)** TBUT; **(D)** OSDI; and **(E)** Oxford corneal staining scores. FES, floppy eyelid syndrome; TBUT, tear break-up time; OSDI, ocular surface disease index.

No study was identified as having a potential source of heterogeneity in the sensitivity analysis (Figure 7). The publication bias was determined using the Egger’s linear regression based on the *p* values and 95% CIs of the prevalence of FES (*p* = 0.475; 95% CI, −7.92 to 15.57), Schirmer 1 value (*p* = 0.082; 95% CI, −7.77 to 0.50), TBUT (*p* = 0.390; 95% CI, −6.00 to 2.42), OSDI (*p* = 0.406; 95% CI, −1.87 to 0.78), and Oxford corneal staining scores (*p* = 0.09; 95% CI, −1.95 to 21.19). Figure 8 shows the findings that were determined to be unaffected by the publication bias.

## 4 Discussion

The eye is a critical organ responsible for vision in humans. The ocular surface performs an indispensable function in the refraction and defense of the eye. The ocular surface anatomy includes corneal epithelium, conjunctiva, and appendages associated with the stabilization of the tear film (Kanellopoulos et al., 2012; Lin et al., 2019). OSAHS is closely associated with damage to several body systems, such as cardiovascular, endocrine, neurological, and ocular surfaces (Lu et al., 2017). This study reported a higher prevalence of OSAHS among patients with eyelid laxity syndrome and dry eye (40 and 48%, respectively). In addition, patients with OSAHS experienced significant changes in the dry eye parameters. Higher OSAHS severity was associated with worsening of dry eye metrics, and the absence of the lid gland was more pronounced in patients with OSAHS. However, there were no substantial differences in the IOP between patients with and without OSAHS. Additionally, the effect of OSAHS severity on the changes in the ocular surface parameters was further analyzed. The AHI values were negatively correlated with the values of Schirmer 1 and TBUT, but positively correlated with the OSDI scores.

Over the past 25 years, an increasing number of studies have investigated ocular surface disorders in patients with OSAHS, with a primary emphasis on dry eye, eyelids, and ocular surface. Cheong et al. (2023) revealed a positive association between OSAHS and eyelid laxity syndrome, and the increase in OSAHS severity was linked to a significant elevation in the risk of FES. Our findings indicate that the patients with OSAHS have a greater risk of developing concomitant FES. Patients who suffer from OSAHS and FES have lower levels of elastic fibers in specific organs of the body, which highlights a probable association between both conditions. This phenomenon provides evidence that the underlying processes could be closely related, even though these mechanisms manifest themselves as two distinct medical conditions. Biological studies in patients with OSAHS and FES have revealed a significant reduction in the number of elastic fibers in certain areas of the body, such as the zygomatic plate, eyelid skin, and uvula (Wang et al., 2016; Nijjar et al., 2022). A previous study has shown that the concentration of the elastic fiber was low in the eyelids and orbicularis oculi muscles of individuals with FES (Idowu et al., 2019). Similarly, the elastin fiber content was significantly low in the uvula and other parts of the pharynx in the patients with OSAHS (Series et al., 2004). Degradation in the elastin fiber is linked to the upregulation of matrix metalloproteinases (MMPs). As per the findings of a previous report, patients with FES have considerably low levels of elastin fiber, which has been associated with higher concentrations of MMPs in the eyelids (Schlotzer-Schrehardt et al., 2005), which in turn lead to the laxity of the eyelids. In apnea and hypopnea, patients with OSAHS experience hypoxic events, which contribute to ischemia-reperfusion damage and enhance oxidative stress due to the enhanced formation of reactive oxygen species (Passali et al., 2015; Stanek et al., 2021). A positive association between MMP overexpression and OSAHS was also been revealed in a meta-analysis by Franczak et al. (2019). Thus, OSAHS promotes the degradation of elastin fibers that results in eyelid laxity via angiogenesis, sympathetic stimulation, systemic and vascular inflammatory processes, and endothelial dysfunction. These findings further justify the biological rationale for the higher rate of combined FES in patients with OSAHS.

Dry eyes develop when tear hypertonicity from evaporation causes damage to the ocular surface. This may happen either directly or indirectly by promoting inflammation (Kamoi et al., 2011). Patients with OSAHS often suffer from dry eyes due to inflammation (Acar et al., 2013; Wang et al., 2016). Pu et al. (2022) confirmed that the prevalence of dry eyes in patients with OSAHS was 42.2% and reached even higher levels—as high as 67.5%—in patients with poor sleep quality. The prevalence of dry eyes in patients with OSAHS in the present study was 48%, which is consistent with previous findings. The ocular surface assessment methods for dry eyes in this study included Schirmer 1 test, TBUT, OSDI, and Oxford corneal staining scores. Overall, the Schirmer 1 and TBUT values were dramatically reduced in patients with OSAHS, whereas the OSDI and Oxford Corneal Staining Score values were substantially increased, particularly in patients with severe disease. Therefore, OSAHS may be a risk factor for dry eyes, and the severity of dry eyes may be correlated with that of OSAHS. In patients with hyperosmolar inflammation, apoptosis of the corneoconjunctival epithelium and cup cells is more likely to occur, which in turn worsens the tear film instability. In addition, tear film instability and tear hyperosmolarity induce inflammation that can lead to a vicious cycle of chronic inflammation of neurogenic origin, and thus increase disease severity (Beckman et al., 2020; Wu et al., 2021; Kasetsuwan et al., 2022). Exposure to intermittent hypoxia in patients with OSAHS leads to systemic inflammatory response and elevated levels of proinflammatory cytokines, such as interleukins 1, 6, and 18 and tumor necrosis factor (Carneiro-Barrera et al., 2022). Cytokines that are generated as a result of injured epithelial cells and dilated conjunctival arteries are the main cause underlying prolonged inflammation. In patients with OSAHS, the increase in the AHI is accompanied by an increase in mechanical stress, hypoxia rates, and ocular surface inflammatory processes. This leads to greater irritation of the lacrimal gland, which in turn leads to diminished corneal sensitivity and attenuated tear secretion. Furthermore, the loss of the lid gland and culet cell function deteriorates the tear film quality and contributes to further exacerbation of the dry eye condition. Two very recent meibography studies have provided evidence that MGD can be implicated in the pathophysiology of OSAHS-related dry eyes (Karaca et al., 2019; Muhafiz et al., 2020). Karaca et al. (2019) reported that patients with severe OSAHS had significantly higher upper eyelid meiboscore values than those having normal snoring patterns, which is in line with the findings of our investigation. These results suggest that ocular surface diseases in patients with OSAHS involve not only tear disorders but also eyelid problems.

In this study, the intraocular pressure (IOP) of the patients with OSAHS was not significantly different than that of the healthy controls. Geyer et al. (2003) observed a correlation between the AHI, glaucoma, and the IOP. Moreover, a study by Mavigok et al. (2022) showed that the included population did not have OSAHS associated with glaucoma, in which typical visual field defects and retinal nerve fiber layer analysis involvement were not observed. In their study, the IOP was in the normal range in both the cases and control groups. On the other hand, Mehta et al. (2022) showed that the association between OSA and glaucoma was influenced by ethnicity, with Malays having a twofold increased risk of glaucoma if they had an intermediate or higher risk of OSA. Lee et al. (2022) observed a correlation between OSAHS and primary closed-angle glaucoma, but not primary open-angle glaucoma. Glaucoma may have many different forms, each of which results in a distinct patient group and a set of diagnostic criteria. The relationship between OSAHS and glaucoma is still a controversial issue. Therefore, the findings of this study have to be further confirmed by expanding the sample size and conducting subgroup analysis for the different types of glaucoma.

In our study, the degree of ocular surface alterations was associated with the severity of OSAHS. As described in the Results section, the AHI values increased and the indicators of ocular surface examination worsened with increase in the number of episodes of apnea and hypoxia. Acar et al. (2013) and Kadyan et al. (2010) measured the TBUT values and evaluated the severity of alterations to the ocular surface using the Schirmer test. According to their findings, the Schirmer values were inversely associated with the TBUT values in terms of OSAHS severity, where a comparatively lower level of tear film was observed in patients with severe OSAHS than in healthy controls. Increased severity of OSAHS leads to more severe nocturnal intermittent hypoxia and increased severity of FES in the affected patients. The clinical symptoms of the patients are characterized primarily by ocular irritation and worsening of dry eyes.

Compared with previously published meta-analyses, this study included recently published high-quality studies, which in turn yields more reliable results. Second, previous reviews have emphasized the systematic evaluation of the association between ocular surface disease and the risk of developing OSAHS. However, the quantitative data from the ocular surface assessment in this review are subjected to combined analysis that lead to more intuitive and refined results. In addition, our findings illustrate a higher rate of comorbid conditions of FES and dry eyes in patients with OSAHS. Therefore, clinicians should consider the ocular surface changes in patients when evaluating OSAHS. According to the present study results, we recommend that patients with severe OSAHS should undergo ocular surface examination at the ophthalmology department. Longitudinal evaluation of ocular surface changes and tear film performance, which include MG dysfunction and cornea fluorescein staining, is required in patients with OSAHS. Accordingly, OSAHS should be considered by ophthalmologists when diagnosing patients who present with dry eye or MG damage of unknown etiology. Overall, our findings are valuable for the early intervention and treatment of combined ocular surface disorders in patients with OSAHS.

However, there are a few limitations to the current study. Covariates like age, sex, and body mass index were not considered in the analytical model used in this study, which could contribute to heterogeneity. Furthermore, many of the included studies did not control for these relevant confounding factors. For instance, age is a crucial confounding factor. As one gets older, the number of elastin fibers decreases, and the lipid metabolism becomes less efficient (De Gregorio et al., 2021). The differences observed between the different age groups might affect the results. However, the participants in all the included studies were adults. The mean age of most participants was in the range of 38–55 years. We could not detect significant differences in the ocular surface assessment outcomes among the different age groups. Thus, it is necessary to conduct further research to verify whether OSAHS can affect the ocular surface disease evaluation when including more children and older adults. As a result of the high prevalence of obesity among people who have OSAHS and ocular surface illness, obesity is considered to be a confounding factor for OSAHS and ocular surface abnormalities (Mastrota, 2008; Bayat et al., 2022). Therefore, the presence of OSAHS and ocular surface disease may only be a collateral phenomenon related to obesity in these patients, and there could be no actual association between OSAHS and ocular surface alterations. Moreover, in the included studies, OSAHS was diagnosed using a variety of approaches, which may affect the accuracy of the true OSAHS sample size.

## 5 Conclusion

As per the findings of this meta-analysis, patients who have OSAHS have a remarkably increased risk of developing FES and dry eye. Additionally, patients with OSAHS usually present with ocular surface alterations. In these patients, early detection and treatment of ocular surface lesions could prevent severe and potentially irreversible ocular surface disease. Accordingly, additional in-depth future studies are required to identify whether there is a link between the risk of OSAHS and alterations in the ocular surface.

## Data Availability

The original contributions presented in the study are included in the article/Supplementary Material. Further inquiries can be directed to the corresponding author.

## References

[B1] Abusharha A. El-Hiti G. A. Alsubaie M. H. Munshi A. F. Alnasif A. R. Fagehi R. (2022). Evaluation of tear evaporation rate in patients with diabetes using a hand-held evaporimeter. Healthc. (Basel) 10, 104. 10.3390/healthcare10010104 PMC8775361 35052268

[B2] Acar M. Firat H. Acar U. Ardic S. (2013). Ocular surface assessment in patients with obstructive sleep apnea-hypopnea syndrome. Sleep. Breath. 17, 583–588. 10.1007/s11325-012-0724-0 22664770

[B3] Acar M. Firat H. Yuceege M. Sanal Dogan A. Caliskan S. Gurdal C. (2018). The presence of conjunctivochalasis in obstructive sleep apnea patients. Eye Contact Lens 44 (1), S163–S166. 10.1097/ICL.0000000000000361 28099284

[B4] Alanazi M. A. El-Hiti G. A. Al-Tamimi R. Bawazir A. M. Almutleb E. S. Fagehi R. (2022). Assessment of the effect of wearing a surgical face mask on tear film in normal eye subjects. J. Ophthalmol. 2022, 2484997. 10.1155/2022/2484997 36017483 PMC9398824

[B5] Baudouin C. Messmer E. M. Aragona P. Geerling G. Akova Y. A. Benitez-del-Castillo J. (2016). Revisiting the vicious circle of dry eye disease: A focus on the pathophysiology of meibomian gland dysfunction. Br. J. Ophthalmol. 100, 300–306. 10.1136/bjophthalmol-2015-307415 26781133 PMC4789719

[B6] Bayat A. H. Aydemir E. Aydemir G. A. Gencer H. (2022). Assessment of tear film anomalies in childhood obesity. Klin. Monbl Augenheilkd 239, 331–337. 10.1055/a-1668-0276 34911123

[B7] Beckman K. Katz J. Majmudar P. Rostov A. (2020). Loteprednol etabonate for the treatment of dry eye disease. J. Ocul. Pharmacol. Ther. 36, 497–511. 10.1089/jop.2020.0014 32391735 PMC7482125

[B8] Bi X. D. Zhao J. Liu J. W. (2022). Analysis of dry status of sleep apnea hypopnea syndrome. J. Clin. Ophthalmol. 30, 425–429. 10.3969/j.issn.1006-8422.2022.05.009

[B9] Bindi I. Ori M. Marchegiani M. Morreale M. Gallucci L. Ricci G. (2022). Diagnosis of upper airways collapse in moderate-to-severe OSAHS patients: A comparison between drug-induced sleep endoscopy and the awake examination. Eur. Arch. Otorhinolaryngol. 279, 2167–2173. 10.1007/s00405-021-07184-8 34839405

[B10] Bitners A. C. Arens R. (2020). Evaluation and management of children with obstructive sleep apnea syndrome. Lung 198, 257–270. 10.1007/s00408-020-00342-5 32166426 PMC7171982

[B11] Bonacci E. Fasolo A. Zaffanello M. Merz T. Brocoli G. Pietrobelli A. (2022). Early corneal and optic nerve changes in a paediatric population affected by obstructive sleep apnea syndrome. Int. Ophthalmol. 42, 1281–1287. 10.1007/s10792-021-02115-2 34738205

[B12] Brautaset R. L. Rosén R. Cerviño A. Miller W. L. Bergmanson J. Nilsson M. (2015). Comparison of macular thickness in patients with keratoconus and control subjects using the cirrus HD-OCT. Biomed. Res. Int. 2015, 832863. 10.1155/2015/832863 26167503 PMC4475698

[B13] Carneiro-Barrera A. Amaro-Gahete F. J. Guillen-Riquelme A. Jurado-Fasoli L. Saez-Roca G. Martin-Carrasco C. (2022). Effect of an interdisciplinary weight loss and lifestyle intervention on obstructive sleep apnea severity: The INTERAPNEA randomized clinical trial. JAMA Netw. Open 5, e228212. 10.1001/jamanetworkopen.2022.8212 35452108 PMC9034401

[B14] Chen L. Liu M. Bao J. Xia Y. Zhang J. Zhang L. (2013). The correlation between apparent diffusion coefficient and tumor cellularity in patients: A meta-analysis. PLoS One 8, e79008. 10.1371/journal.pone.0079008 24244402 PMC3823989

[B15] Cheong A. J. Y. Ho O. T. W. Wang S. K. X. Woon C. Y. Yap K. Ng K. J. Y. (2023). Association between obstructive sleep apnea and floppy eyelid syndrome: A systematic review and metaanalysis. Surv. Ophthalmol. 68, 257–264. 10.1016/j.survophthal.2022.11.006 36427560

[B16] Cristescu T. R. Mihaltan F. D. (2020). Ocular pathology associated with obstructive sleep apnea syndrome. Rom. J. Ophthalmol. 64, 261–268. 10.22336/rjo.2020.43 33367159 PMC7739556

[B17] De Gregorio A. Cerini A. Scala A. Lambiase A. Pedrotti E. Morselli S. (2021). Floppy eyelid, an under-diagnosed syndrome: A review of demographics, pathogenesis, and treatment. Ther. Adv. Ophthalmol. 13, 25158414211059247. 10.1177/25158414211059247 35187400 PMC8855428

[B18] Deng X. Tian L. Zhang Y. Li A. Cai S. Zhou Y. (2022). Is histogram manipulation always beneficial when trying to improve model performance across devices? Experiments using a meibomian gland segmentation model. Front. Cell Dev. Biol. 10, 1067914. 10.3389/fcell.2022.1067914 36544900 PMC9760981

[B19] Dhillon S. Shapiro C. M. Flanagan J. (2007). Sleep-disordered breathing and effects on ocular health. Can. J. Ophthalmol. 42, 238–243. 10.3129/canjophthalmol.i07-029 17392846

[B20] Fiedorczuk P. Polecka A. Walasek M. Olszewska E. (2023). Potential diagnostic and monitoring biomarkers of obstructive sleep apnea-umbrella review of meta-analyses. J. Clin. Med. 12, 60. 10.3390/jcm12010060 PMC9821668 36614858

[B21] Fox T. P. Schwartz J. A. Chang A. C. Parvin-Nejad F. P. Yim C. K. Feinsilver S. H. (2017). Association between eyelid laxity and obstructive sleep apnea. JAMA Ophthalmol. 135, 1055–1061. 10.1001/jamaophthalmol.2017.3263 28880982 PMC5710483

[B22] Franczak A. Bil-Lula I. Sawicki G. Fenton M. Ayas N. Skomro R. (2019). Matrix metalloproteinases as possible biomarkers of obstructive sleep apnea severity - a systematic review. Sleep. Med. Rev. 46, 9–16. 10.1016/j.smrv.2019.03.010 31060030

[B23] Fukuda M. Sasaki H. (2012). Quantitative evaluation of corneal epithelial injury caused by n-heptanol using a corneal resistance measuring device *in vivo* . Clin. Ophthalmol. 6, 585–593. 10.2147/opth.S30935 22553418 PMC3340121

[B24] Garcia-Sanchez A. Villalain I. Asencio M. Garcia J. Garcia-Rio F. (2022). Sleep apnea and eye diseases: Evidence of association and potential pathogenic mechanisms. J. Clin. Sleep. Med. 18, 265–278. 10.5664/jcsm.9552 34283018 PMC8807908

[B25] Geyer O. Cohen N. Segev E. Rath E. Z. Melamud L. Peled R. (2003). The prevalence of glaucoma in patients with sleep apnea syndrome: Same as in the general population. Am. J. Ophthalmol. 136, 1093–1096. 10.1016/s0002-9394(03)00709-8 14644220

[B26] Grover D. P. (2010). Obstructive sleep apnea and ocular disorders. Curr. Opin. Ophthalmol. 21, 454–458. 10.1097/ICU.0b013e32833f00dc 20811281

[B27] Gunes I. Oltulu R. Oltulu P. Turk N. Yosunkaya S. (2023). Ocular surface in patients with obstructive sleep apnea syndrome: Evaluation of clinical parameters and impression cytology. Eye Contact Lens 49, 14–18. 10.1097/ICL.0000000000000945 36138005

[B28] Hwang H. B. Ku Y. H. Kim E. C. Kim H. S. Kim M. S. Hwang H. S. (2020). Easy and effective test to evaluate tear-film stability for self-diagnosis of dry eye syndrome: Blinking tolerance time (BTT). BMC Ophthalmol. 20, 438. 10.1186/s12886-020-01686-5 33148200 PMC7640480

[B29] Idowu O. O. Ashraf D. C. Vagefi M. R. Kersten R. C. Winn B. J. (2019). Floppy eyelid syndrome: Ocular and systemic associations. Curr. Opin. Ophthalmol. 30, 513–524. 10.1097/ICU.0000000000000617 31483320

[B30] Kadyan A. Asghar J. Dowson L. Sandramouli S. (2010). Ocular findings in sleep apnoea patients using continuous positive airway pressure. Eye (Lond) 24, 843–850. 10.1038/eye.2009.212 19680276

[B31] Kamoi M. Ogawa Y. Uchino M. Tatematsu Y. Mori T. Okamoto S. (2011). Donor-recipient gender difference affects severity of dry eye after hematopoietic stem cell transplantation. Eye (Lond) 25, 860–865. 10.1038/eye.2011.73 21475315 PMC3178168

[B32] Kanellopoulos A. J. Aslanides I. M. Asimellis G. (2012). Correlation between epithelial thickness in normal corneas, untreated ectatic corneas, and ectatic corneas previously treated with CXL; is overall epithelial thickness a very early ectasia prognostic factor? Clin. Ophthalmol. 6, 789–800. 10.2147/OPTH.S31524 22701079 PMC3373227

[B33] Karaca E. E. Akcam H. T. Uzun F. Ozdek S. Ulukavak Ciftci T. (2016). Evaluation of ocular surface health in patients with obstructive sleep apnea syndrome. Turk J. Ophthalmol. 46, 104–108. 10.4274/tjo.57778 27800271 PMC5076291

[B34] Karaca I. Yagci A. Palamar M. Tasbakan M. S. Basoglu O. K. (2019). Ocular surface assessment and morphological alterations in meibomian glands with meibography in obstructive sleep apnea Syndrome. Ocul. Surf. 17, 771–776. 10.1016/j.jtos.2019.06.003 31226420

[B35] Kasetsuwan N. Suwan-Apichon O. Lekhanont K. Chuckpaiwong V. Reinprayoon U. Chantra S. (2022). Assessing the risk factors for diagnosed symptomatic dry eye using a smartphone app: Cross-sectional study. JMIR Mhealth Uhealth 10, e31011. 10.2196/31011 35731569 PMC9260529

[B36] Kim Y. H. Oh T. W. Park E. Yim N. H. Park K. I. Cho W. K. (2018). Anti-inflammatory and anti-apoptotic effects of acer palmatum thumb. Extract, KIOM-2015ew, in a hyperosmolar-stress-induced *in vitro* dry eye model. Nutrients 10, 282. 10.3390/nu10030282 29495608 PMC5872700

[B37] Kohler M. (2015). Deleterious systemic effects of OSA: How much evidence do we need? Thorax 70, 817–818. 10.1136/thoraxjnl-2015-207247 26173952

[B38] Kohler M. Stradling J. R. (2010). Mechanisms of vascular damage in obstructive sleep apnea. Nat. Rev. Cardiol. 7, 677–685. 10.1038/nrcardio.2010.145 21079639

[B70] Lee J. Choi H. J. (2021). Accuracy and reliability of measurements obtained with a noncontact tono-pachymeter for clinical use in mass screening. Sci. Rep. 11, 8900. 10.1038/s41598-021-88364-8 33903678 PMC8076298

[B39] Lee S. Y. Yu H. Kim D. K. (2022). Glaucoma is associated with the risk of obstructive sleep apnea: A population-based nationwide cohort study. Diagn. (Basel) 12, 2992. 10.3390/diagnostics12122992 PMC9776797 36552999

[B40] Lin H. Liu Y. Yiu S. (2019). Three dimensional culture of potential epithelial progenitor cells in human lacrimal gland. Transl. Vis. Sci. Technol. 8, 32. 10.1167/tvst.8.4.32 PMC6716802 31523489

[B41] Lin P. W. Lin H. C. Chang C. T. Friedman M. Salapatas A. M. Lin M. C. (2022). Alterations of ocular surface and tear film in patients with obstructive sleep apnea/hypopnea syndrome. Nat. Sci. Sleep. 14, 277–290. 10.2147/NSS.S340105 35450223 PMC9017596

[B42] Lin X. Xu B. Zheng Y. Coursey T. G. Zhao Y. Li J. (2017). Meibomian gland dysfunction in type 2 diabetic patients. J. Ophthalmol. 2017, 3047867. 10.1155/2017/3047867 28593054 PMC5448054

[B43] Liu M. Gao Y. Y. (2017). Ten years of achievements in biological and medical sciences. Chin. J. Optom. Ophthalmol. Vis. Sci. 19, 111–115. 10.1007/s11427-017-9003-3 28215028

[B44] Liu S. Li S. Li M. Zeng S. Chen B. Zhang L. (2022). Evaluation of the ocular surface and meibomian gland in obstructive sleep apnea hypopnea syndrome. Front. Med. (Lausanne) 9, 832954. 10.3389/fmed.2022.832954 35223929 PMC8863666

[B45] Lu D. Li N. Yao X. Zhou L. (2017). Potential inflammatory markers in obstructive sleep apnea-hypopnea syndrome. Bosn. J. Basic Med. Sci. 17, 47–53. 10.17305/bjbms.2016.1579 27754829 PMC5341778

[B46] Ma X. R. Wang Y. Sun Y. C. (2019). Imbalance of osteoprotegerin/receptor activator of nuclear factor-kappa B ligand and oxidative stress in patients with obstructive sleep apnea-hypopnea syndrome. Chin. Med. J. 132, 25–29. 10.1097/cm9.0000000000000046 30628956 PMC6629297

[B47] Mastrota K. M. (2008). Impact of floppy eyelid syndrome in ocular surface and dry eye disease. Optom. Vis. Sci. 85, 814–816. 10.1097/OPX.0b013e3181852777 18772717

[B48] Mavigok E. Ozcan A. A. Ulas B. (2022). Obsructive sleep apnea syndrome: Is it a risk factor for ocular surface disease and ocular comorbidities? Int. Ophthalmol. 10.1007/s10792-022-02629-3 36580155

[B49] McCann U. D. Sgambati F. P. Schwartz A. R. Ricaurte G. A. (2009). Sleep apnea in young abstinent recreational MDMA ("ecstasy") consumers. Neurology 73, 2011–2017. 10.1212/WNL.0b013e3181c51a62 19955499 PMC2790228

[B50] Mehta A. Man R. E. K. Gan A. T. Najjar R. P. Nongpiur M. Lamoureux E. L. (2022). Association between risk of obstructive sleep apnea and glaucoma: The Singapore epidemiology of eye diseases study. J. Glaucoma 31, 935–940. 10.1097/IJG.0000000000002105 35980862

[B51] Muhafiz E. Olcen M. Erten R. Bozkurt E. (2020). Evaluation of meibomian glands in obstructive sleep apnea-hypopnea syndrome. Cornea 39, 685–690. 10.1097/ICO.0000000000002252 31939920

[B52] Nijjar M. Kotoulas S. C. Kerr J. Riha R. L. (2022). Floppy eyelid syndrome and obstructive sleep apnea: A unique phenotype? Sleep. Breath. 10.1007/s11325-022-02690-3 35943691

[B53] Passali D. Corallo G. Yaremchuk S. Longini M. Proietti F. Passali G. C. (2015). Oxidative stress in patients with obstructive sleep apnoea syndrome. Acta Otorhinolaryngol. Ital. 35, 420–425. 10.14639/0392-100X-895 26900248 PMC4755047

[B54] Phillips M. E. Fowler B. T. Dryden S. C. Fleming J. C. (2019). Canthal V-plasty for floppy eyelid surgery. Plast. Reconstr. Surg. Glob. Open 7, e2464. 10.1097/gox.0000000000002464 31772892 PMC6846288

[B55] Pu Q. Wu Z. Li A. L. Guo X. X. Hu J. J. Li X. Y. (2022). Association between poor sleep quality and an increased risk of dry eye disease in patients with obstructive sleep apnea syndrome. Front. Med. (Lausanne) 9, 870391. 10.3389/fmed.2022.870391 36388897 PMC9659957

[B56] Schlotzer-Schrehardt U. Stojkovic M. Hofmann-Rummelt C. Cursiefen C. Kruse F. E. Holbach L. M. (2005). The pathogenesis of floppy eyelid syndrome: Involvement of matrix metalloproteinases in elastic fiber degradation. Ophthalmology 112, 694–704. 10.1016/j.ophtha.2004.11.031 15808264

[B57] Series F. Chakir J. Boivin D. (2004). Influence of weight and sleep apnea status on immunologic and structural features of the uvula. Am. J. Respir. Crit. Care Med. 170, 1114–1119. 10.1164/rccm.200404-458OC 15306538

[B58] Stanek A. Brozyna-Tkaczyk K. Myslinski W. (2021). Oxidative stress markers among obstructive sleep apnea patients. Oxid. Med. Cell Longev. 2021, 9681595. 10.1155/2021/9681595 34336121 PMC8321764

[B59] Stang A. (2010). Critical evaluation of the Newcastle-Ottawa scale for the assessment of the quality of nonrandomized studies in meta-analyses. Eur. J. Epidemiol. 25, 603–605. 10.1007/s10654-010-9491-z 20652370

[B60] Stansbury R. C. Strollo P. J. (2015). Clinical manifestations of sleep apnea. J. Thorac. Dis. 7, E298–E310. 10.3978/j.issn.2072-1439.2015.09.13 26543619 PMC4598518

[B61] Tomic M. Kastelan S. Soldo K. M. Salopek-Rabatic J. (2013). Influence of BAK-preserved prostaglandin analog treatment on the ocular surface health in patients with newly diagnosed primary open-angle glaucoma. Biomed. Res. Int. 2013, 603782. 10.1155/2013/603782 23971041 PMC3732629

[B62] Ulutas H. G. Balikci Tufekci A. Gunes A. (2022). Evaluation of corneal, ocular surface, and meibomian gland changes in obstructive sleep apnea syndrome. J. Fr. Ophtalmol. 45, 191–200. 10.1016/j.jfo.2021.09.007 34961649

[B63] Vaccaro O. Imperatore G. Ferrara A. Palombino R. Riccardi G. (1992). Epidemiology of diabetes mellitus in southern Italy: A case-finding method based on drug prescriptions. J. Clin. Epidemiol. 45, 835–839. 10.1016/0895-4356(92)90066-v 1624965

[B64] Vakil M. Park S. Broder A. (2018). The complex associations between obstructive sleep apnea and auto-immune disorders: A review. Med. Hypotheses 110, 138–143. 10.1016/j.mehy.2017.12.004 29317057

[B65] Veloso J. F. Oriá A. P. Raposo A. C. S. Lacerda A. J. Silva C. V. B. Lima L. F. (2020). The use of tear ferning test in cats for evaluation of ocular surface. Acta Vet. Scand. 62, 23. 10.1186/s13028-020-00523-5 32456655 PMC7248460

[B66] Wang P. Yu D. J. Feng G. Long Z. H. Liu C. J. Li H. (2016). Is floppy eyelid syndrome more prevalent in obstructive sleep apnea syndrome patients? J. Ophthalmol. 2016, 6980281. 10.1155/2016/6980281 27366328 PMC4913017

[B67] Wang X. H. You W. Wu Z. M. Wu X. Q. Ye F. (2020). Apnea hypopnea index is an independent predictor of coronary microcirculatory dysfunction in stable angina pectoris patients with a single borderline lesion. Acta Cardiol. Sin. 36, 207–215. 10.6515/acs.202005_36(3).20190923a 32425435 PMC7220967

[B68] Wang Y. Huang S. Yu P. (2019). Association between circulating neuregulin4 levels and diabetes mellitus: A meta-analysis of observational studies. PLoS One 14, e0225705. 10.1371/journal.pone.0225705 31815951 PMC6901220

[B69] Wu Y. Wang C. Wang X. Mou Y. Yuan K. Huang X. (2021). Advances in dry eye disease examination techniques. Front. Med. (Lausanne) 8, 826530. 10.3389/fmed.2021.826530 35145982 PMC8823697

